# Quality, Reliability, and Dissemination of In Vitro Fertilization–Related Videos on Chinese Social Media: Cross-Sectional Analysis of 300 Short Videos

**DOI:** 10.2196/83900

**Published:** 2026-01-28

**Authors:** Xueyan Bai, Feng Guo, Dapeng Chu

**Affiliations:** 1 Beijing Chao-Yang Hospital, Capital Medical University, Main Campus, Beijing Chao-Yang Hospital, 8 Gongren Tiyuchang Nanlu, Chaoyang District, Beijing, 100020, Beijing, 10020, China, 86 01085231000; 2 APUCH Innovation, Beijing, China

**Keywords:** in vitro fertilization, social media, health communication, content quality, misinformation

## Abstract

**Background:**

Patients increasingly rely on short-video platforms for information regarding in vitro fertilization (IVF), yet the relationship between the scientific quality of this content and its algorithmic dissemination remains unclear.

**Objective:**

This study aimed to assess the quality, reliability, and key drivers of dissemination of IVF-related short videos on major Chinese social media platforms.

**Methods:**

A cross-sectional content analysis was conducted on 300 popular IVF-related videos (the top 100 results from each platform) retrieved from Douyin, Bilibili, and Xiaohongshu between January 10 and 15, 2025. Video quality and reliability were evaluated using the Global Quality Score and a modified DISCERN instrument. Predictors of video dissemination were identified using an Extreme Gradient Boosting machine learning model, with the number of “likes” serving as the primary outcome variable.

**Results:**

Content produced by medical professionals demonstrated significantly higher quality and reliability (median mDISCERN 11.0, IQR 9.0-15.0) compared to non-medical sources (median mDISCERN 8.0, IQR 5.0-13.0; *P*< .001). However, the Extreme Gradient Boosting analysis identified the uploader’s follower count as the most powerful predictor of video “likes.” In contrast, quality metrics (Global Quality Score and modified DISCERN scores) had a negligible impact on dissemination.

**Conclusions:**

In the current Chinese social media landscape, the dissemination of IVF-related videos is strongly associated with creator influence rather than scientific merit. This disconnect between engagement and quality poses a potential risk of misinformation, highlighting the need for medical professionals to adopt platform-native communication strategies to ensure that high-quality information reaches patients.

## Introduction

In vitro fertilization (IVF) offers profound hope to individuals and couples facing infertility, yet the journey is fraught with challenges. The complexity of the procedures, significant financial costs, and uncertain outcomes impose a substantial physiological, psychological, and economic burden on patients [1]. In navigating this demanding process, access to accurate, comprehensive, and understandable medical information is critical for informed decision-making, managing treatment expectations, and mitigating psychological distress [2-5]. Historically, this information was primarily disseminated by health care institutions. However, the digital era has precipitated a paradigm shift, with patients increasingly turning to the internet for more accessible and diverse sources of support [6-8]. While social media’s impact on health behaviors is a global phenomenon, China’s digital ecosystem offers a unique context for study. With the world’s largest internet user base and high demand for assisted reproductive technology, platforms such as Douyin (the Chinese counterpart to TikTok) provide a critical “natural laboratory” to understand algorithmic health communication patterns that are increasingly relevant worldwide.

In recent years, short-video platforms such as Douyin, Bilibili, and Xiaohongshu have emerged as dominant arenas for health information dissemination, distinguished by their algorithm-driven, highly engaging, and rapidly propagating nature [9,10]. While these platforms present unprecedented opportunities for medical education, they also introduce formidable challenges [11-13]. Unlike traditional medical websites, content generation is often spontaneous and lacks rigorous professional oversight, creating a “perfect storm of information” where quality is highly variable [14,15]. Furthermore, their personalized recommendation algorithms, while enhancing user experience, risk creating “information cocoons” that can amplify biased or inaccurate content [16,17], posing a potential hazard to patients seeking IVF treatment, especially concerning misinformation on reproductive health [18-20].

Despite growing analyses of social media health content, the IVF domain on Chinese short-video platforms remains understudied. Moreover, prior work has largely assessed quality in isolation, leaving unclear whether intrinsic quality or extrinsic platform factors (eg, creator influence) primarily drive dissemination. The few studies that have explored dissemination dynamics have relied on conventional linear models, which are ill-equipped to capture the complex, nonlinear factors driving content virality in sophisticated social networks. This leaves our understanding of the contemporary health information ecosystem fundamentally incomplete.

To address this gap, we conducted a cross-sectional analysis of the top-ranked IVF videos on Douyin, Bilibili, and Xiaohongshu. Our methodological approach consisted of three phases: (1) content analysis to classify uploader identity and topics, (2) assessment of information quality using the Global Quality Score (GQS) and the modified DISCERN (mDISCERN) instrument, and (3) machine learning analysis using Extreme Gradient Boosting (XGBoost) and Shapley Additive Explanations (SHAP) values to isolate independent predictors of video dissemination among metadata variables. We hypothesized that uploader influence (follower count), rather than content quality, would be the dominant predictor of engagement. The findings are intended to provide an evidence-based foundation for enhancing the effective communication of high-quality medical information and to offer actionable guidance for platforms, content creators, and public health authorities.

## Methods

### Study Design and Video Retrieval

A cross-sectional study was designed to evaluate the quality, reliability, and dissemination of IVF-related videos across three popular Chinese social media platforms: Xiaohongshu, Bilibili, and Douyin [21]. These platforms were selected based on their market dominance and distinct demographic profiles. Douyin (Chinese version of TikTok, (ByteDance, Beijing, China), with 766 million daily active users (DAUs) as of 2024 [22], represents China’s short-video mainstream platform, with videos typically ranging from 15 to 60 seconds in length [23], Bilibili (Bilibili Inc., Shanghai, China), with an average of 104 million DAUs in 2024 [24], specializes in medium-to-long form video content (typically 3‐30 min), with medium and long videos accounting for 70% of platform views [25]. The platform’s user base is predominantly young, with nearly 70% of China’s Generation Z population and an average user age of 25 years [24]. Xiaohongshu (RedNote, Xiaohongshu, Shanghai, China), with 143 million global DAUs by the end of 2024 [26], serves as a lifestyle-focused platform with a predominantly female user base (70% female) and functions as a primary search engine for lifestyle and health-related decisions among Chinese women [27].

Using the Chinese keyword “试管婴儿” (IVF), relevant videos were retrieved from each platform between January 10 and 15, 2025. To mitigate the influence of personalized recommendations, searches were conducted using newly created accounts with no prior viewing history. No filters or sorting mechanisms were applied, thereby simulating a typical user experience.

An initial systematic search on the 3 platforms identified 531 potentially relevant videos. These records were then screened for eligibility based on predefined inclusion and exclusion criteria. A total of 231 videos were excluded for the following reasons: duplicate content (n=127, 55.0%), non–Chinese-language content (n=14, 6.1%), being purely promotional without educational value (n=55, 23.8%), or having content irrelevant to the topic of IVF (n=35, 15.2%). This screening process yielded a final sample of 300 (56.5%) unique videos for analysis, comprising the top 100 eligible videos from each platform.

We used a quota sampling strategy based on platform search rankings. For each platform, videos were retrieved and screened sequentially, starting from the top-ranked search result. The screening process continued down the ranked list until a quota of 100 eligible videos meeting all inclusion and exclusion criteria was reached for each platform. This strategy ensures that the sample reflects the content most visible to users, as search rankings prioritize high-engagement content. These rankings are algorithmically driven and prioritize a synthesis of user engagement metrics (eg, likes, comments, and shares), topical relevance, and content freshness, thereby simulating the ecological search experience of a typical user. Selection was subject to the following criteria: (1) the video was in the Chinese language, (2) the video focused on IVF-related medical content, and (3) the video was publicly accessible. Videos were excluded if they contained (1) duplicate content, (2) pure advertisements without educational value, (3) videos unrelated to IVF, and (4) non–Chinese-language videos. For each video, basic information was documented, including title, upload date, duration, uploader identity, and engagement metrics (eg, likes, comments, shares, and saves). Regarding cross-platform posting, videos uploaded by the same creator to multiple platforms were treated as distinct analytical units. This approach was chosen because engagement metrics (eg, likes and comments) are platform specific and reflect the unique algorithmic distribution and audience reaction within that specific ecosystem. However, intraplatform duplicates (the same video uploaded twice to the same platform) were excluded.

The diagram illustrates the selection process for the study. Initially, a keyword search for “试管婴儿” (IVF) was conducted on 3 platforms, namely, Douyin, Bilibili, and Xiaohongshu, identifying 231, 500, and 390 videos, respectively. Following the platforms’ comprehensive ranking algorithms, the top-ranked videos were selected for screening (Douyin, n=116; Bilibili, n=120; and Xiaohongshu, n=122). During the screening phase, videos were excluded for being duplicates or thematically irrelevant (Douyin, n=16; Bilibili, n=20; and Xiaohongshu, n=22). Finally, 100 eligible videos from each platform were included, resulting in a total of 300 videos for the final analysis.

### Video Classification

Videos were categorized by uploader and content type by 2 independent researchers, with disagreements resolved by a third reviewer, following established content analysis methodologies [28,29].

All included videos were systematically classified according to a predefined coding scheme focusing on two primary dimensions: uploader identity and content theme. The uploader of each video was categorized into one of five groups: medical professionals, which included verified IVF doctors, reproductive medicine institutions, or health care providers; health science communicators or key opinion leaders, defined as individuals known for disseminating health knowledge without formal medical credentials; patients and sharers, consisting of individuals sharing personal IVF treatment experiences; marketing promoters, identified as commercial entities promoting fertility services; and news and general content creators, such as media outlets providing general information.

Concurrently, the primary subject matter of each video was assigned to one of five content categories: medical knowledge, comprising scientific explanations, clinical guidelines, or technical information; fertility and lifestyle optimization, focusing on content related to lifestyle practices intended to enhance fertility; patient experience sharing, which covered personal narratives detailing individual IVF journeys; policy and ethical topics, which included discussions on regulations or social implications; and misleading or marketing content, which included promotional material or videos with verifiably inaccurate information.

### Quality and Reliability Assessment

Video quality and reliability were assessed using the GQS [30,31] and the mDISCERN instrument [32,33].

The GQS is a 5-point scale evaluating overall quality, flow, and integrity of information (1=poor and 5=excellent) and has been validated in numerous studies of web-based health information [30,31]. The mDISCERN instrument was adapted from the original 16-item DISCERN tool [32,33] to specifically evaluate short-form digital health content. To accommodate the brevity of social media videos, the instrument was condensed into five core dimensions:

1. Reliability of information: assesses the evidentiary basis and accuracy of medical claims
2. Clarity of aims: evaluates whether the video’s purpose and structure are clearly communicated
3. Relevance of sources: measures the transparency and authority of cited evidence (eg, clinical guidelines vs unverifiable anecdotes)
4. Balance and impartiality: assesses the extent of commercial bias or one-sided promotion
5. Presentation of uncertainty: evaluates the disclosure of risks, side effects, and biological variability

Items 9 to 15 of the original DISCERN tool, which focus on detailed treatment choices and shared decision-making, were excluded as they are rarely applicable to brief, nonconsultative video clips. Furthermore, the scoring system was modified from the original 1- to 5-point scale to a 0- to 5-point Likert scale. This modification allowed for a score of “0” to explicitly categorize content that was completely devoid of sources, reliability, or clear aims, which is a common characteristic of low-quality user-generated content.

Two experienced reproductive medicine specialists independently scored all videos. Initial disagreements were resolved through discussion to reach a consensus. If a consensus could not be reached, the score from the third senior specialist was used as the final arbitration score. Interrater reliability was assessed and found to be high (Cohen κ coefficient >0.80), indicating a strong degree of agreement [34,35].

### Dissemination Analysis With XGBoost

An XGBoost regression model was used to identify factors influencing video dissemination, with the number of “likes” as the primary outcome variable [36,37]. Input features included platform, uploader category, content category, GQS score, mDISCERN score, video length, days since publishing, and the presence of background music or subtitles. To investigate the primary drivers of raw engagement, the model was first trained on the untransformed “likes” count.

Furthermore, to account for the highly skewed distribution of the “likes” variable and to build a more stable model for confirming feature contributions, a secondary analysis was performed using a logarithmic transformation (log1p) on the target variable before model training. This standard preprocessing technique helps mitigate the influence of outlier videos with extremely high engagement.

Hyperparameter tuning was performed using grid search with 3-fold cross-validation to optimize model performance [38,39]. The importance of features was assessed using SHAP values to provide transparent and intuitive insights into how each factor influences dissemination [40,41].

### Statistical Analysis

All statistical analyses were conducted using R (version 4.2.3; R Foundation for Statistical Computing). Owing to the nonnormal distribution, nonparametric tests were used, including the Kruskal-Wallis test for multigroup comparisons and the Dunn test for post-hoc analysis. The Spearman correlation coefficient was used to assess relationships between variables. Statistical significance was defined as *P*<.05.

### Ethical Considerations

This study analyzed publicly available, user-generated IVF-related videos and their metadata from Chinese social media platforms. The research involved no interaction or intervention with individuals and did not collect, store, or process identifiable personal data. In accordance with institutional and national guidelines, the use of publicly accessible, aggregate data does not constitute human subjects research; therefore, ethics approval and consent to participate were not required.

## Results

### Overview

**Figure 1.**
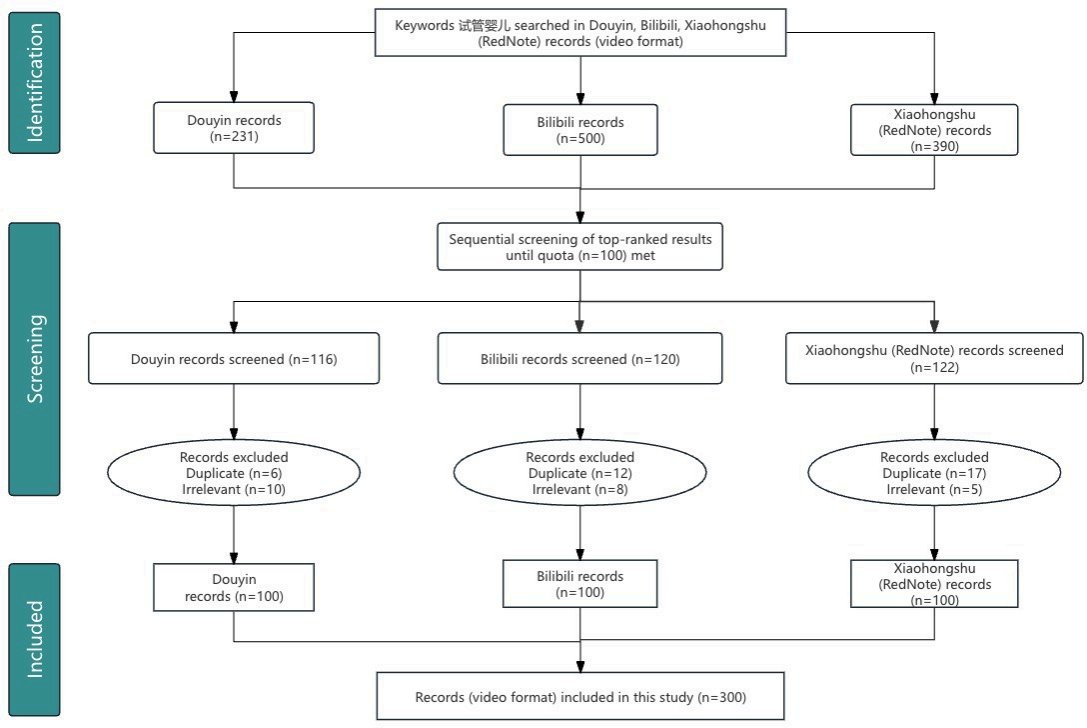
Flow diagram of the video identification, screening, and inclusion process.

The complete selection process is detailed in the video selection flow diagram (Figure 1). The study’s findings reveal a fundamental paradox in the digital health ecosystem: while the quality of IVF-related information is critically dependent on the professional identity of its creator, its dissemination is overwhelmingly governed by the creator’s platform influence, not the quality of the content itself.

### Platform Ecosystems Exhibit Profound Heterogeneity

Analysis of 300 videos revealed significant heterogeneity in content strategy, user base, and engagement dynamics across platforms (*P*<.001; Table 1). On Douyin and Xiaohongshu, medical professionals were the dominant uploaders (87/100, 87%, and 89/100, 89% respectively), and content was primarily “medical knowledge” (71/100, 71% on both). In contrast, Bilibili featured a more diverse creator base, including patient sharers (28/100, 28%) and health science communicators (22/100, 22%), with “patient experience sharing” (31/100, 31%) being more prevalent (Table 2).

**Table 1.** Baseline of in vitro fertilization–relevant videos.

Characteristics	Douyin (n=100)	Bilibili (n=100)	Xiaohongshu (n=100)	*P* value
Likes, median (IQR)	539 (81‐2986.5)	138 (16‐844.5)	277.5 (66‐925.25)	<.001
Saves, median (IQR)	145 (11-732)	78 (12-415)	134 (40‐525.75)	.31
Comments, median (IQR)	30 (6.75‐327.25)	17 (3‐149.5)	51 (7.75‐126.5)	.16
Shares, median (IQR)	161 (9.5‐785)	56 (5-310)	96.5 (26.5‐437.75)	.10
Days since uploading, median (IQR)	116 (100.75‐155)	788.5 (293.25‐1300.75)	218.5 (125‐394.25)	<.001
Length, median (IQR)	41.5 (25.75‐59)	325 (169-692)	53 (37.75‐94)	<.001
Followers, median (IQR)	36000 (7469.25‐273,500)	4679.5 (322.25‐35,500)	8769.5 (3010.75‐33,000)	<.001
Total video count, median (IQR)	364 (167-560)	201 (66.5‐488.5)	309.5 (191.25‐758.25)	.004
Type of video, n				<.001
Medical knowledge	71	43	71	
Fertility & lifestyle optimization	0	10	3	
Patient experience sharing	19	31	18	
Policy & ethical topics	8	12	3	
Misleading or marketing content	2	4	5	
Type of uploader, n				<.001
Medical professionals	87	24	89	
Health science communicators/medical key opinion leaders (KOLs)	1	22	0	
Patients and fertility journey sharers	7	28	5	
Marketing promoters	1	11	6	
News and general interest content creators	4	15	0	
BGM^a^, n				<.001
Without BGM	38	46	19	
With BGM	62	54	81	
Subtitle, n				.02
Without subtitle	0	6	1	
With subtitle	99	94	99	
GQS^b^ score, median (IQR)	2 (2-3)	2 (2-3)	3 (2-3)	.04
DISCERN score, median (IQR)	10 (7-14)	10 (6‐14.25)	12 (9-15)	.02

a BGM: background music.

b GQS: Global Quality Score.

**Table 2.** Characteristics of uploaders and video content across the 3 platforms (N=300).

Category	Douyin (n=100), n (%)	Bilibili (n=100), n (%)	Xiaohongshu (RedNote) (n=100), n (%)	*P* value
Uploader profile				<.001
Medical professionals	87 (87)	24 (24)	89 (89)	
Patient voices	1 (1)	28 (28)	5 (5)	
Health communicators or KOLs^a^	7 (7)	22 (22)	6 (6)	
Marketing and promoters	4 (4)	11 (11)	0 (0)	
News and general content	1 (1)	15 (15)	0 (0)	
Video content type				<.001
Medical knowledge	71 (71)	43 (43)	71 (71)	
Patient experience	19 (19)	31 (31)	18 (18)	
Fertility and lifestyle	8 (8)	12 (12)	3 (3)	
Policy and ethics	0 (0)	10 (10)	3 (3)	
Misleading and marketing	2 (2)	4 (4)	5 (5)	

a KOL: key opinion leader.

Video attributes also differed significantly. Bilibili hosted older (median 788.5, IQR 293.3-1300.8 d) and longer videos (median 325, IQR 169-692 s), whereas Douyin featured more recent, shorter-form content (median 41.5, IQR 25.8-59 s; *P*<.001 for both). Douyin creators had the largest median follower counts (n=36,000) and generated the highest median “likes” (n=539), significantly outperforming the other platforms. These baseline differences in creator influence and content strategy precede the analysis of dissemination drivers.

### Quality and Reliability Assessment

Overall, video quality and reliability were moderate. Interrater agreement for the scoring was high (weighted κ_GQS_=0.82, 95% CI 0.76‐0.87; weighted κ_mDISCERN_=0.79, 95% CI 0.72‐0.85; International Code Council_GQS_=0.90, 95% CI 0.86‐0.93; International Code Council_mDISCERN_=0.88, 95% CI 0.84‐0.91).

**Figure 2.**
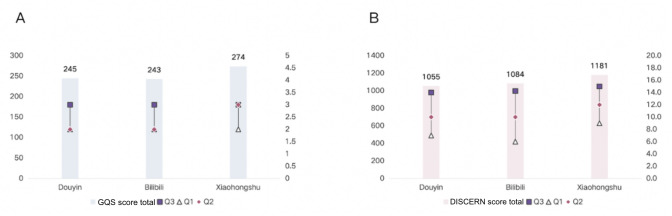
Comparison of Global Quality Score (GQS) and DISCERN score distributions across 3 platforms. (A) Distribution of GQS scores across the 3 platforms; (B) distribution of DISCERN scores across the 3 platforms.

Platform-level analysis showed that Xiaohongshu videos achieved a statistically significant higher quality, with a median GQS of 3.0 (IQR 2.0-3.0; *P*=.04) and a median mDISCERN score of 12.0 (IQR 9.0-15.0; *P*=.02) compared to Douyin and Bilibili (Figure 2).

**Figure 3.**
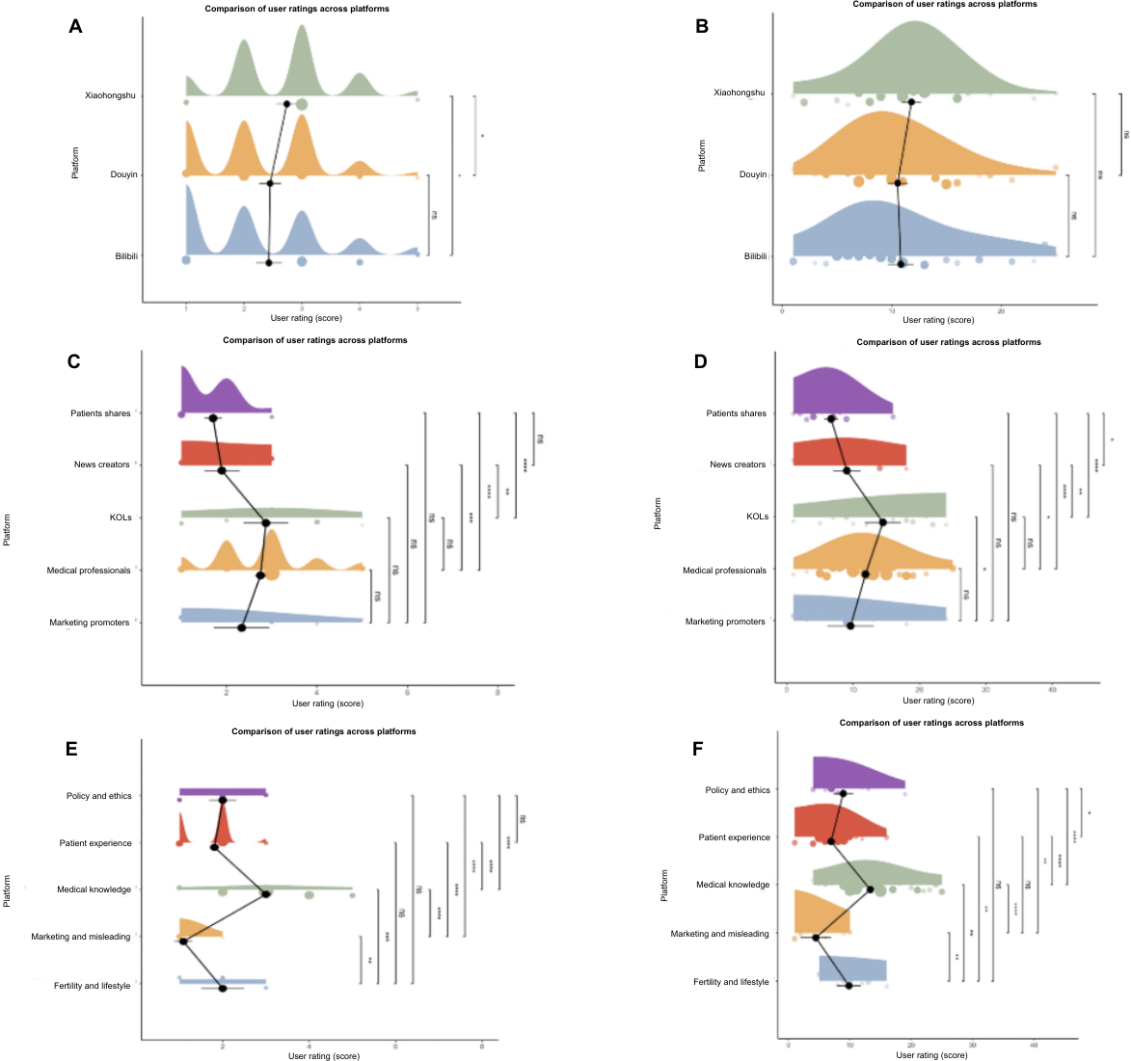
Comparison of video quality and reliability scores across different subgroups. (A) Distribution of Global Quality Score (GQS) scores across the 3 platforms; (B) distribution of DISCERN scores across the 3 platforms; (C) distribution of GQS scores across the 5 uploader types; (D) distribution of DISCERN scores across the 5 uploader types; (E) distribution of GQS scores across the 5 content types; and (F) distribution of DISCERN scores across the 5 content types. Each subplot displays the data distribution using a violin plot and a box plot. Asterisks indicate the level of statistical significance from pairwise statistical tests. *P<.05, **P<.01, ***P<.001. KOL: key opinion leader.

Subgroup analysis revealed a strong association between uploader identity and content quality (*P*<.001). Our data highlights a distinct “competence hierarchy”: videos from medical professionals consistently achieved the highest reliability scores (median mDISCERN 11.0, IQR 9.0-15.0), reflecting adherence to clinical guidelines. Conversely, patient sharers and marketing promoters scored significantly lower. While patient sharers provide emotional value, their content often lacked medical accuracy (median GQS 2.0, IQR 1.0-2.0), suggesting that the “lived experience” often comes at the expense of clinical precision. Videos categorized as “medical knowledge” were rated significantly higher than all other content themes (*P*<.001; Figure 3).

### Predictors of Video Dissemination

To resolve the disconnect between content creation and consumption, an XGBoost machine learning model was developed to identify the primary drivers of video dissemination (“likes”). Initial bivariate correlation analysis found no significant relationship between a video’s like count and its GQS or mDISCERN scores, providing a preliminary suggestion that quality was not a key factor for engagement.

**Figure 4.**
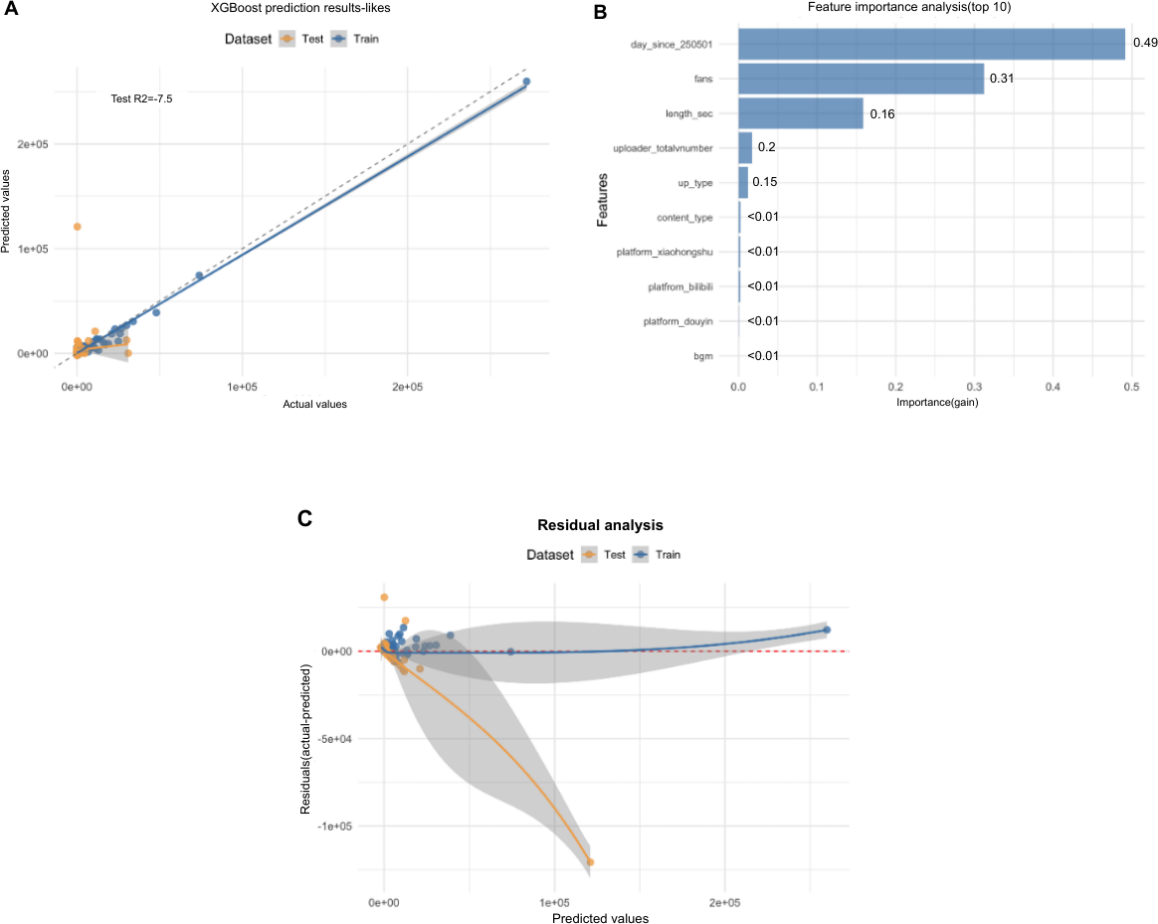
Results of the XGBoost model interpreting the factors influencing video likes: (A) the goodness of fit of the XGBoost model (*R*^2^=−7.5); (B) the importance of each variable on the number of video likes; and (C) the Shapley Additive Explanation summary plot showing the impact of important variables on the model output. XBoost: Extreme Gradient Boosting.

The initial XGBoost model, trained directly on the untransformed “likes” count, yielded a negative *R*^2^ of −7.5 (Figure 4A). This result confirms that raw social media engagement follows a nonlinear, heavy-tailed distribution that cannot be modeled by standard additive regression. Consequently, feature importance rankings from this initial model were disregarded to avoid spurious conclusions (Figure 4). We therefore relied exclusively on the secondary log1p-transformed model for identifying predictors.

A closer examination of the SHAP summary plot (Figure 4) clarifies how these top features influence predictions. For the “fans” feature, a clear positive trend is visible: high feature values (represented by red dots) are strongly associated with high positive SHAP values, confirming that a larger follower count directly contributes to a higher prediction of likes. In contrast, for metrics such as GQS and mDISCERN, the points are clustered vertically around the zero-line with no discernible color gradient, visually confirming their negligible impact on the model’s output and reinforcing the quality-impact gap.

**Figure 5.**
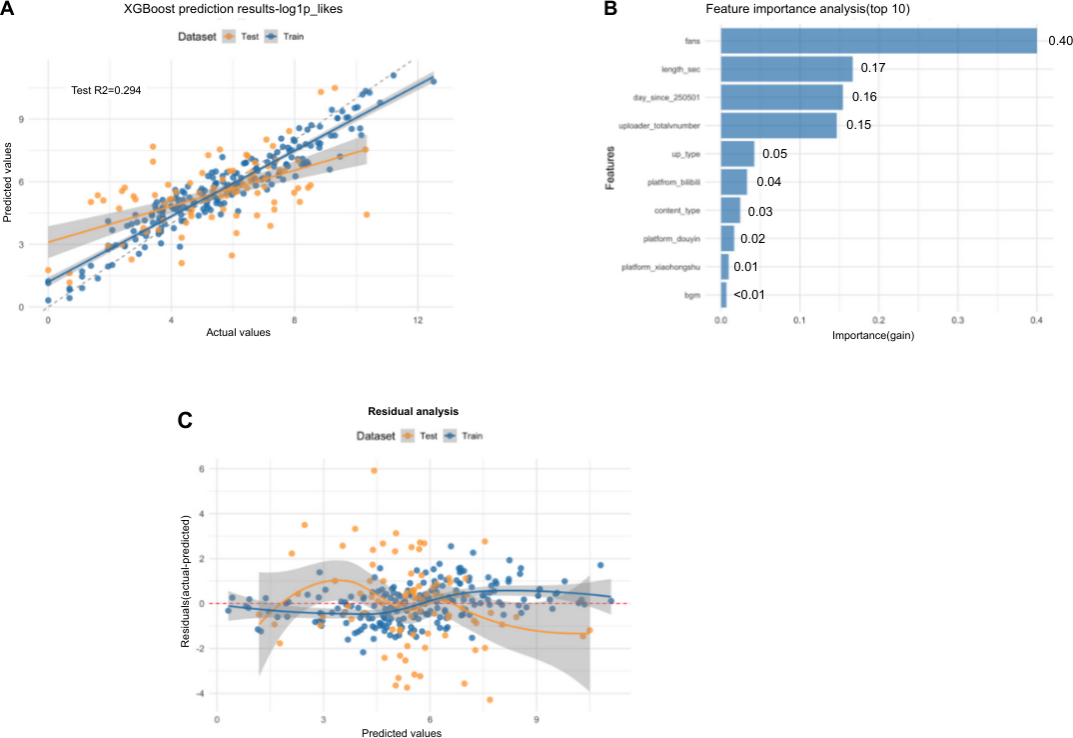
Performance and interpretation of the XGBoost model for predicting video likes. (A) Prediction results of the XGBoost model, plotting actual versus predicted values for the log1p_likes target. The coefficient of determination for the test set is *R*^2^=0.294. (B) Feature importance analysis showing the top 10 predictors ranked by their gain score. (C) Residual analysis plotting residuals against predicted values to assess model fit and error distribution. XGBoost: Extreme Gradient Boosting.

To ensure these findings were not an artifact of the target variable’s extreme skewness and to robustly validate the feature hierarchy, we developed a second XGBoost model by applying a logarithmic transformation (log1p) to the “likes” count. This standard data preprocessing step resulted in a more stable model with a positive predictive fit, achieving an *R*^*2*^ of 0.294 on the test set (Figure 5A). The corresponding residual plot confirmed a more balanced and desirable error distribution (Figure 5C).

Crucially, despite the improved model performance, the feature importance analysis remained remarkably consistent (Figure 5B). The uploader’s follower count was once again the most dominant predictor. In stark contrast, the validated metrics for content quality and reliability—GQS and mDISCERN scores—remained at the bottom of the feature importance hierarchy, exerting negligible influence. This dual-model approach provides robust, 2-fold evidence that in the current algorithmic landscape, a video’s reach is driven primarily not by its scientific merit but by the preexisting social capital of its creator.

## Discussion

### The “Quality-Impact Gap” in Digital Health

This study provides the first systematic evaluation of IVF-related health information on major Chinese short-video platforms, revealing a significant and troubling paradox at the heart of the modern digital health landscape. Our analysis empirically demonstrates a divergence between content quality and engagement. While quality metrics (GQS and mDISCERN) track closely with medical expertise, dissemination metrics (likes) track with uploader influence. This suggests that high-quality medical information does not automatically generate high engagement. This “quality-impact gap” [42] implies that scientific accuracy is not the primary driver of algorithmic visibility. This phenomenon is not merely an algorithmic quirk; it reflects a fundamental tension between the clinical nature of information and the socioemotional needs of patients. Patients navigating the arduous IVF journey are not just passive consumers of data; they are actively seeking hope, validation, and a sense of community. Consequently, low-quality but emotionally resonant content—such as unverified “miracle baby” testimonials—may be perceived as more valuable than dry, technically accurate explanations, leading to higher engagement.

Clinically, this gap risks therapeutic misconception and spending on unproven add-ons among IVF patients. To mitigate harm, clinicians and fertility centers should coproduce platform-native content—short, narrative-driven videos that embed evidence (success rates and indications or contraindications), use on-screen references, and include myth-fact segments—and deploy them via verified accounts with regular posting cadence and call-to-action links to authoritative resources [11,43].

Our central finding—that an uploader’s follower count is the most potent predictor of reach—must be interpreted with nuance. In the fast-paced digital environment, follower count acts as a powerful cognitive heuristic for trust. Lacking the time or expertise to critically appraise every video, users subconsciously substitute “popularity” for “credibility,” operating under the assumption that a large following implies authority and trustworthiness [43]. This dynamic, where social capital eclipses scientific capital, aligns perfectly with findings from Western platforms such as YouTube [44,45] and extends recent analyses of other medical topics on Chinese platforms [46,47]. Our research confirms that this algorithmic prioritization of engagement over evidence is a universal feature of contemporary social media architecture [48,49], creating a global challenge for evidence-based health communication.

### Platform Ecosystems: Expert-Led Versus Community-Driven

Furthermore, our 3-platform comparison revealed distinct “platform personalities” that shape this information flow. Douyin and Xiaohongshu function primarily as expert-led, knowledge-dissemination channels, yet they are still subject to the influencer dynamic. In contrast, Bilibili operates as a community-driven, experience-sharing hub (with 28% patient sharers vs ≤7% on other platforms), hosting longer narratives that fulfill patients’ documented need for peer support [50]. While valuable for emotional well-being, this “experiential” content was found to be of significantly lower quality, posing a risk of normalizing anecdotal advice over clinical guidelines.

The implications of this ecosystem for patient care and public health are profound. For a vulnerable population already facing immense emotional and financial stress, the stakes are exceptionally high. Exposure to misinformation or low-quality content can foster therapeutic misconceptions, leading patients to pursue unproven and costly adjunct therapies. It can create unrealistic expectations about success rates, leading to deeper psychological distress when treatments fail. Moreover, the prevalence of marketing content masquerading as educational material exposes patients to potential financial exploitation [51]. The “attention economy” of social media is thus not a neutral marketplace of ideas; for IVF patients, it is a high-risk environment where the most visible information is often the least reliable.

### Clinical Implications and Future Directions

Therefore, a paradigm shift is imperative for medical professionals and health care organizations. A passive approach of simply producing high-quality content and expecting it to be discovered is destined to fail. A proactive, 2-pronged strategy is required. First, proactive content creation demands that clinicians become platform-native communicators [11,52,53]. This means moving beyond static informational videos and embracing storytelling, patient-centered narratives, and visually compelling formats developed in collaboration with communication experts, without compromising scientific integrity [54]. Second, reactive engagement is equally crucial. Medical professionals and institutions should consider themselves “digital first responders,” actively identifying and correcting high-reach misinformation through comments, response videos, or collaborations with platforms—a practice shown to be effective in other health contexts [43].

Looking forward, a clear agenda for future research emerges from this work. While our quantitative model identified what drives dissemination, qualitative studies are now needed to understand why. In-depth interviews with IVF patients could illuminate the specific motivations and cognitive processes behind their engagement with different types of content. Furthermore, interventional research is urgently needed to design and test the efficacy of novel communication strategies. Randomized controlled trials could compare the reach and impact of standard informational videos against narrative-based, emotionally resonant, yet scientifically accurate content. Finally, longitudinal studies are required to track the real-world impact of social media exposure on patient decision-making, treatment adherence, and clinical outcomes over time.

This study has several notable strengths. It is the first to systematically analyze IVF content across China’s 3 dominant short-video platforms. By using a robust content analysis methodology underpinned by validated instruments—GQS and the mDISCERN tool with high interrater reliability (κ>0.80)—we provided a rigorous assessment of content quality. Methodologically, our use of a dual XGBoost modeling strategy provides a particularly robust analysis. We first demonstrated the model’s inability to predict absolute “likes” (*R*^2^=−7.5), empirically confirming the highly stochastic nature of social media virality [55]. We then solidified our conclusions by using a second, logarithmically transformed model, which, despite a better predictive fit (*R*^*2*^=0.294), reproduced the exact same feature importance hierarchy. This confirmatory step ensures that our central finding is robust and not an artifact of data skewness. Third, while our optimized XGBoost model achieved a robust *R*^2^ of 0.294, approximately 70% of the variance in dissemination remains unexplained. This suggests that engagement is influenced by unmeasured “soft” factors extrinsic to medical quality or uploader status, such as thumbnail aesthetics, opening “hooks” (the first 3 s of video), emotional delivery, and platform-specific trending audio. Future studies should use computer vision and sentiment analysis to quantify these variables.

### Limitations

However, the study’s limitations must be acknowledged. First, our sampling strategy restricted analysis to the top-ranked videos. While this design validly represents the “information diet” of a typical user who rarely scrolls beyond the first page, it introduces selection bias. Our findings characterize the most visible content ecosystem rather than the entire universe of IVF-related videos. Additionally, its cross-sectional design precludes any causal inference. The findings are specific to the Chinese social media context and may not be generalizable. Finally, our analysis used “likes” as the primary proxy for dissemination. Future research could conduct a more granular analysis exploring the distinct drivers of other interaction types, such as comments or shares, which may reflect different dimensions of user engagement [56].

### Conclusions

In conclusion, our research paints a stark picture of the IVF information landscape on Chinese social media, where the mechanisms of dissemination are dangerously decoupled from the principles of evidence-based medicine. Our dual-model analysis robustly demonstrates that the digital health ecosystem does not inherently reward quality; it rewards influence. To bridge the gap between what is popular and what is reliable, the medical community must not only produce trustworthy information but also master the art and science of platform-native communication to ensure that their expertise can successfully navigate the algorithmic currents and reach the patients who need it most.

## Abbreviations

DAU: daily active user
GQS: Global Quality Score
IVF: in vitro fertilization
mDISCERN: modified DISCERN
SHAP: Shapley Additive Explanations
XGBoost: Extreme Gradient Boosting
