# Correction: Quality, Reliability, and Dissemination of In Vitro Fertilization–Related Videos on Chinese Social Media: Cross-Sectional Analysis of 300 Short Videos

**DOI:** 10.2196/95354

**Published:** 2026-04-22

**Authors:** Xueyan Bai, Feng Guo, Dapeng Chu

**Affiliations:** 1 Beijing Chao-Yang Hospital, Capital Medical University, Main Campus, Beijing Chao-Yang Hospital, 8 Gongren Tiyuchang Nanlu, Chaoyang District, Beijing, 100020, Beijing, 10020, China, 86 01085231000; 2 APUCH Innovation, Beijing, China

In “Quality, Reliability, and Dissemination of In Vitro Fertilization–Related Videos on Chinese Social Media: Cross-Sectional Analysis of 300 Short Videos” [1], the authors made a change to the order of authorship.

The authorship order was originally presented as:

Dapeng Chu, Xueyan Bai, Feng Guo

This has been changed to the following:

Xueyan Bai, Feng Guo, Dapeng Chu

The correction will appear in the online version of the paper on the JMIR Publications website, together with the publication of this correction notice. Because this was made after submission to PubMed, PubMed Central, and other full-text repositories, the corrected article has also been resubmitted to those repositories.
